# The relationship between different iodine sources and nutrition in pregnant women and adults

**DOI:** 10.3389/fendo.2022.924990

**Published:** 2022-07-26

**Authors:** Rong Sun, Lijun Fan, Yang Du, Lanchun Liu, Tingting Qian, Meng Zhao, Wenjing Che, Peng Liu, Dianjun Sun

**Affiliations:** ^1^ Center for Endemic Disease Control, Chinese Center for Disease Control and Prevention, Harbin Medical University, Harbin, China; ^2^ Key Laboratory of Etiology and Epidemiology, Education Bureau of Heilongjiang Province (23618504) and Ministry of Health, Microelement and Human Health Laboratory of Heilongjiang Province, Center for Endemic Disease Control, Chinese Center for Disease Control and Prevention, Harbin Medical University, Harbin, China

**Keywords:** iodine supplement, measure, nutrition, pregnant women, adults

## Abstract

**Background:**

Different iodine supplement measures emerge along with the economy development in China. The article objectives are to compare and explore the relationship between iodine sources and nutrition of pregnant women and adults.

**Methods:**

A total of 2,145 pregnant women and 1,660 adults were investigated by multi-stage random method. Questionnaire was used to collect basic information and the consumption of food, water, and iodine preparations. Household salt and individual urine and blood samples were collected, and thyroid function and morphology of pregnant women were measured.

**Results:**

The median urinary iodine concentration (MUIC) of pregnant women (164.49 μg/L) was lower than adults (187.30 μg/L, *p* < 0.05). Iodine supplement with IS (iodized salt) was the main measure for pregnant women and adults, and the difference was mainly on the consumption of iodine preparations between pregnant women (5.19%) and adults (0.85%). Moreover, adults’ dietary iodine intake from food (100.6 μg/day), IS (140.8 μg/day), and drinking water (6.0 μg/day) was higher than those of pregnant women (86.5, 107.2, and 3.5 μg/day, respectively). Compared with iodine supplement with IS, ISFP (IS + iodine-rich food + iodine preparations) could reduce the risk of iodine deficiency for pregnant women. The MUICs for pregnant women and adults of iodine supplements with IF (iodine-rich food) and ISF (IS + iodine-rich food) were lower. For pregnant women, thyroid nodule (11.90%) and peroxidase antibody (TPOAb) positive (9.32%) were high prevalent thyroid diseases, and habitation (urban/rural), gestation, annual income, and drinking water type would affect them.

**Conclusion:**

Pregnant women and adults had adequate iodine nutrition in four provinces. Their iodine supplement measures were different, the consumption of iodine preparations in pregnant women was higher, and their dietary iodine intake was lower than adults. ISFP was an effect measure for pregnant women to supplement iodine.

## Introduction

Iodine is one of the essential trace elements in human body (1), which can only be obtained from the diet. After iodine entering the human body through the mouth, it could be quickly and completely absorbed into the plasma in the stomach and the upper segment of the small intestine. In the thyroid, most of the iodine is absorbed and concentrated by thyroid follicular epithelial cells from plasma, whereas the iodine without being absorbed and utilized is mainly excreted through the kidney by urine. Thyroid is the organ with the strongest ability to absorb and concentrate iodine (2). Iodine acts on many organs through synthetic thyroid hormones, regulating metabolism, promoting growth, and so on (3). Iodine deficiency (ID) or excess will have a certain impact on human health (4, 5). Insufficient iodine intake will lead to ID disorders (IDDs) represented by endemic goiter and cretinism, and iodine excess also will result in thyroid abnormality such as hypothyroidism and subclinical hypothyroidism. Since 1995, according to the recommendation of WHO, China has implemented the Universal Salt Iodization strategy, IDD has been eliminated in 28 out of 31 provinces by the year 2010 (6). However, recently, some thyroid diseases had been found rise again (7), which lead to a heated discussion about the iodine supplement and the intake amount (8–10).

With the development of society, iodized salt (IS) is still an effect method to supplement iodine, but it has not been the only measure for population. Many people began to supplement iodine with diet, especially iodine-rich foods (such as kelp and laver) (11, 12). Study has shown that the human body needed iodine, in which more than 80% comes from food, 10%–20% from drinking water, and less than 5% from the air (13). In addition, Ling et al. pointed out that the daily intake of a certain amount of IS and iodine-rich food would complement with each other to improve iodine nutrition (14). However, the content of iodine in food mainly depends on the environment, which would have a dual impact on iodine nutrition (excessive or deficiency). The ocean is rich in iodine, about 50 μg/L. Kelp, seaweed, shellfish, and sea shrimp contain more iodine, but their iodine concentration levels are different (15). Compared with food, the iodine in drinking water is easier to be absorbed and utilized, but the fluctuation of iodine content has a larger dynamic range (from 0.1 to 150 μg/L) (16), so people in areas with high-iodine–containing water are extremely susceptible to excessive iodine intake (17). In some western provinces, to maintain adequate iodine nutrition, iodized oil pills are used to supplement iodine, and iodine-contained multi-vitamin preparations have become a recommended measure by clinicians, especially for pregnant women. Moreover, people’s dietary habits and consumption are also different, which lead to different iodine intake in places; therefore, it is very important to explore appropriate iodine supplement measure.

Pregnant women is a special population, and their iodine intake needs not only to meet their own health needs but also to supply the growth of the fetus (18). Studies have confirmed that pregnant women’s iodine nutrition will directly affect the thyroid function and neurological intelligence development of fetal (19–21). Compared with adults, the hormone level and metabolism of pregnant women have changed, their requirement of iodine is higher, and the adverse consequences caused by ID are more serious (22). Many studies about adults’ thyroid diseases were carried out, but there is still a gap of thyroid diseases prevalence in pregnant women (23). Pregnancy is the period with high prevalence of thyroid diseases, which further illustrates the importance of iodine supplement for them (24).

As the prevalence of thyroid disease increase, more concerning emerged about excessive consumption of IS, and long-term excess salt intake will perhaps lead to cardiovascular, cerebrovascular diseases, etc. The study about salt consumption from 1990 to 2010 found that the per-capita salt intake of 181 countries exceeded the recommended value of WHO (25). Chinese average salt intake was twice of the daily recommended salt intake of WHO (26). In addition, many restaurants, takeout stores, etc., added a lot of condiments containing salt to increase the taste of food, which might also be the reason for excessive iodine intake (27).

This study was carried out for the following purposes: first, to compare the iodine supplement measures and dietary contribution between pregnant women and adults; second, to explore the relationship of iodine supplement measures and nutrition; third, to describe the iodine nutrition and thyroid diseases in four provinces of China and to analyze the influencing factors of pregnant women’s thyroid diseases; and, finally, to investigate their daily salt intake and the dining location of adults.

## Methods

### Investigation sites

A multi-stage random sampling method was adopted. First, four provinces were selected according to their location in China, which were Shanxi (the eastern northern province), Yunnan (the western southern province), Fujian (the eastern southern province, coastal province), and Xinjiang (the western northern province). Some representative geographical areas (counties) were selected in each province (such as historical and non-endemic areas in Yunnan), and high iodine and its surrounding areas were excluded in the choosing field sites; moreover, all the field sites reached the IDD disease elimination standard. Then, every participating county was divided into five sampling areas, east, west, south, north, and middle, according to the requirements of the National Program for Monitoring Iodine Deficiency Disorders. Andersen et al. recommended survey population sample size for the reliability of iodine nutrition surveys (28); the sample size of each group was determined by the variation of the urinary iodine concentration; the number of spot urine samples needed to estimate the iodine level in a population with 90% confidence within a precision range of ±10% was about 86, whereas 400 pregnant women and adults in each province were surveyed in this investigation, which was consistent with 90% confidence within a precision range of ±10% of the iodine nutrition survey.

### Subjects

All the subjects of the investigation should have no special dietary habits. In addition, the subjects of pregnant women should be aged between 20 and 40 years old and were healthy in the past and have no history of diagnosed thyroid diseases, autoimmune diseases, endocrine diseases, and family genetic diseases. As for the subjects of adults, they should age from 18 to 60 years old, and pregnant women and lactating women were excluded.

Other exclusion criteria include the following: ①have a history of smoking or alcohol abuse; ② using iodine-containing preparations in the past 3 days (referred to the iodized oil preparations supplied by government for about once or twice per year in some western provinces), or taken Cydiodine Buccal Tablets or Amiodarone Hydrochloride, and those who had an angiography examination recently; and ③ people with iodine occupational exposure (such as medical personnel who use iodine disinfectant and iodine contrast agent).

### Survey method

In January 2021, the project provinces officially launched the on-site investigation after carrying out different forms of training. First, the provinces recruited respondents; their basic information were obtained by questionnaire; urine, serum, and household salt samples were collected in the local health center and then examined in the provincial laboratory; and thyroid ultrasonic testing was carried out by experienced technician and evaluated by provincial professional. The subjects were screened according to the including criteria and the willingness to participate in the survey of dietary iodine intake.

### Determination method and evaluation standard

The basic information, the history of thyroid diseases, frequency and intake of IS, foods (refers to iodine content more than 10 μg/100 g, and dietary iodine contribution of more than 1%), and preparations were collected through questionnaires. Because of the particularity of pregnant women, only the dining place of adults was surveyed. The household salt, urine, and serum samples were collected for determination of salt iodine, urinary iodine, and thyroid function, respectively. The home salt iodine was measured by direct titration (29), which was strictly followed the procedures, and each sample used three parallel samples. Urinary iodine concentration of spot urine samples was measured using the As^3+^−Ce^4+^catalytic spectrophotometry (30), used two parallel samples, and made the absolute value of the correlation coefficient of the standard curve regression equation more than 0.999. The thyroid volume and function of pregnant women were detected by B-ultrasound and electrochemiluminescence, respectively; thyroid function and B-ultrasound were measured by experienced technicians in provincial center for disease control and prevention using same type instrument to ensure the accuracy.

The pregnant women’s reference range of TSH and FT_4_ were specific, divided by gestation (31, 32): first trimester: TSH, 0.09–4.52 mU/L; FT_4_, 13.15–20.78 pmol/L; middle trimester: TSH, 0.45–4.32 mU/L; FT_4_, 9.77–18.89 pmol/L; and last trimester: TSH, 0.30–4.98 mU/L; FT_4_, 9.04–15.22 pmol/L. The reference range for thyroid antibodies and FT_3_ were as follows: TPOAb, 0–34 IU/ml; TgAb, 0–115 IU/ml; FT_3_, 3.1–6.8 pmol/L. The thyroid volume was calculated according to 0.479 × (left lobe: length × width × thickness + right lobe: length × width × thickness) (length, width, and thickness of each lobe in equation used unit of cm); then, it was judged according to whether the thyroid volume was greater than 18 ml for women and 25 ml for men (33). Thyroid abnormality was defined in this research including hypothyroidism, subclinical hypothyroidism, hyperthyroidism, subclinical hyperthyroidism, TPOAb positive, TgAb positive, double antibody positive, thyroid goiter, and nodule. The labor intensity was classified as light manual labor (work was mainly done sitting or standing, such as office work, cleaning, and child care), medium manual labor (including walking, weeding, playing tennis, dancing, skiing, and biking), and heavy manual labor (including non-mechanized agricultural work, mountain climbing, logging, basketball, and football).

### Statistical analysis

The iodine intake of different sources was calculated according to the frequency and the amount of consumption. Literature (34) and ingredient manual were consulted concerning the content of iodine in various foods (refers to the iodine of the food itself) and preparations, respectively. Iodine content in IS of different household was determined. The subjects of pregnant women and adults were separately divided into five groups concerning to different iodine supplement measures, including iodine-rich food group (IF; refers to eating more than once per month of 50 g or above, wet weight, of kelp, seaweed, etc., and not eating IS), IS group (IS), IS + iodine-rich Food group (ISF), IS + iodine preparations group (ISP; self-report iodine supplement habit for the last year), and IS + iodine-rich food + iodine preparations group (ISFP). The median urinary iodine concentration (MUIC) of each province was calculated, and thyroid functions of pregnant women were determined. Other indicators calculated including the iodine supplement rate (refers to the proportion of subjects receiving iodine supplement through either IS, iodine supplement preparations, or iodine-rich food in all surveyed subjects), iodine supplement contribution (refers to the proportion of iodine intake from drinking water, food, iodine preparations, or IS), consumption rate of qualified IS, detection rate of thyroid disease, etc.

The SPSS 22.0 (produced by International Business Machines Corp) was used for statistical analysis. For normal distribution data, the mean and standard difference were calculated; for skewed distribution data, median and interquartile range were analyzed. Non-parametric rank test was used to compare the MUICs between pregnant women and adults and between different iodine supplement measures. Binary logistic regression was adopted to analyze the effect of different iodine supplement on iodine nutrition. Chi-square test method and Fisher’s exact probability method were used to compare the iodine supplement of pregnant women and adults and to study the influencing factors of pregnant women’s thyroid diseases. All tests were two-side, and *p* < 0.05 was defined as significance.

### Ethics committee approval

This study was conducted according to the guidelines established in the Declaration of Helsinki, and all procedures involving human volunteers were approved by the Ethics Committee of Harbin Medical University (20190502). Written informed consent was obtained from each subject.

## Results

### Basic information of subjects

A total of 2,145 pregnant women and 1,660 adults were investigated, and their basic information was listed (**Table 1**). In terms of pregnant women, first, 812 pregnant women were in urban, accounting for 69.70%. Second, subjects included 380 in the first trimester (17.72%), 1,024 in the middle trimester (47.74%), and 741 in the last trimester (34.54%). Finally, in view of dietary habit, most was three meals a day (73.98%), followed by four meals a day (13.39%), and five or six meals a day (8.37%). As for adults, 759 women (45.72%) and 901 men (54.28%) were investigated in this study. In addition, the number of adults with light manual labor was 809, accounting for 52.70%.

**Table 1:** Basic information of subjects in four provinces.

Type	Indicators	Classification	Number	Proportion (%)
Pregnant Women (n = 2,145)	Habitation	Village	353	30.30
		Urban	812	69.70
	Gestation	First trimester	380	17.72
		Middle trimester	1,024	47.74
		Last trimester	741	34.54
	Degree of education	Primary/junior high school	478	41.07
		Senior high school/junior college	531	45.62
		Bachelor degree or above	155	13.31
	Pregnancy Vomiting reaction	No	727	34.41
		Slight	942	44.58
		Moderate	297	14.06
		Severe	147	6.95
	Dietary habit	Three meals a day	1,564	73.98
		Four meals a day	283	13.39
		Five or six meals a day	177	8.37
		Rule-less	90	4.26
	Drinking water type	Tap water	1302	61.47
		Others	816	38.53
	hypertension	Yes	11	1.17
		No	932	98.83
Adults (n = 1,660)	Gender	Male	759	45.72
		Female	901	54.28
	Labor intensity	Light manual labor	809	52.70
		Medium manual labor	553	36.03
		Heavy manual labor	173	11.27
	Drinking water type	Tap water	1,297	83.19
		Others	262	16.81
	hypertension	Yes	260	15.69
		No	1397	84.31

^a^MUIC, median urinary iodine concentration.

^b^MConsumption rate, the number of subjects receiving qualified iodized salt (iodine preparations or iodine-rich food) / the total number of subjects surveyed.

^c^Iodine supplement rate, the proportion of subjects who take iodized salt, iodine supplement preparations or iodine-rich foods.

^d^Qualified iodized salt, salt samples with iodine of 18-33mg/kg.

### Iodine supplement situation

**Figure 1:**
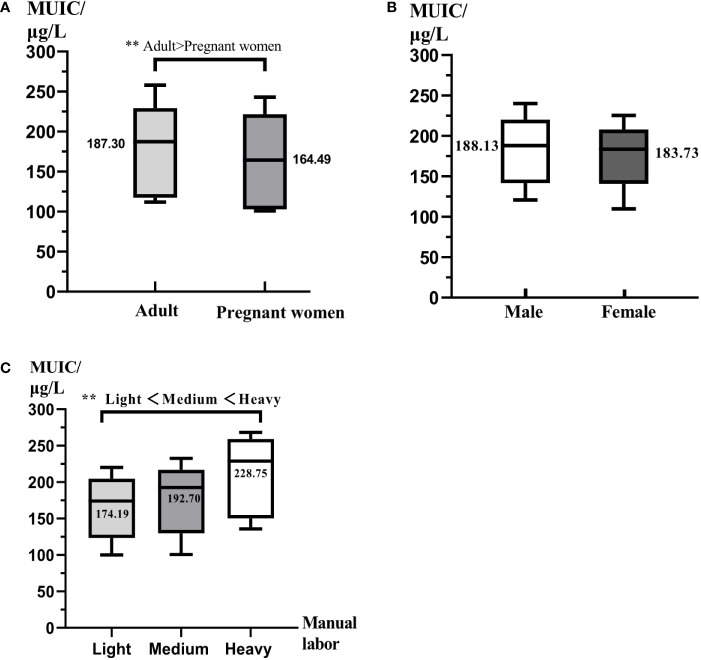
The comparison of iodine nutrition for different populations. All data were presented as median and interquartile range. MUIC was median urinary iodine concentration. **(A)** The comparison of iodine nutrition between adults and pregnant women. **(B)** The comparison of iodine nutrition between men and women. **(C)** The comparison of iodine nutrition of different labor intensity. ** represents P < 0.01.

A total of 2,128 pregnant women and 1,493 adults were examined (**Table 2**). First of all, from the perspective of iodine nutrition, pregnant women with MUIC of 164.49 μg/L were lower than adults (187.30 μg/L) (*p* < 0.05, **Figure 1A**), but all of them were adequate. Adults women and men had the similar iodine nutrition level (**Figure 1B**); moreover, the median urinary iodine of adults with light manual labor was 174.19 μg/L (**Figure 1C**), followed by medium manual labor (192.70 μg/L) and heavy manual labor (228.75 μg/L); all of them had statistic significant (*p* < 0.05). Compared with the iodine nutrition in different provinces, the MUICs of Xinjiang were the highest; pregnant women and adults were 216.21 and 224.28 μg/L, respectively. For pregnant women, the MUIC in Yunnan was the lowest, 138.65 μg/L, followed by Fujian (145.41 μg/L); for adults, the iodine nutrition of Fujian was the lowest, 168.75 μg/L. Then, judging by the iodine supplement rate, the iodine supplement rates for pregnant women in Fujian, Shanxi, Yunnan, and Xinjiang were 100.00%, 98.07%, 100.00%, and 97.80%, respectively, and all the rate of adults were 100%. Finally, in terms of the consumption rate of different iodine supplement, the consumption rates of qualified IS of pregnant women and adults were similar, which were 93.43% and 93.85%, respectively. The consumption rate of qualified IS of pregnant women in Xinjiang was the lowest (78.43%), whereas the lowest rate of adults was in Fujian (89.66%); there was a great difference in the rate of iodine preparations intake between adults and pregnant women, with pregnant women of 5.19%, whereas adults were 0.85%, and even Fujian’s pregnant women had a rate of 10.65%. As for iodine-rich food, the consumption rates of pregnant and adults were similar, and the highest consumption rate was in Fujian (pregnant women, 66.57%; and adults, 53.16%), which was relative to the geographic location (Fujian was a coastal province); however, the lowest consumption rate was in Xinjiang, with 20.82% of pregnant women and 7.83% of adults.

**Table 2:** The iodine supplement situation of adults and pregnant women.

Type	District	Number	MUIC^a^(μg/L)Median(IQR)	Salt Iodine(mg/kg)Mean(SD)	Consumption Rate^b^ (%)			Rate of IodineSupplement^c^
					Qualified Iodine Salt^d^	Iodine Preparations	Iodine-Rich Food	
Pregnant women	Fujian	338	145.41, 100.51–214.70	23.80 ± 5.37	100.00	10.65	66.57	100.00
	Shanxi	874	161.50, 91.21–247.23	25.30 ± 5.43	96.59	1.98	32.51	98.07
	Yunnan	413	138.65, 91.90–192.80	26.01 ± 2.79	98.03	0.98	32.20	100.00
	Xinjiang	503	216.21, 145.65–288.80	27.21 ± 4.14	78.43	7.60	20.82	97.80
	Total	2128	164.49, 104.78–243.00	25.65 ± 4.84	93.43	5.19	35.53	98.56
Adults	Fujian	376	168.75, 103.62–228.73	24.63 ± 2.69	89.66	0.26	53.16	100.00
	Shanxi	345	180.30, 104.10–259.85	25.55 ± 4.87	92.46	2.00	23.25	100.00
	Yunnan	388	186.70, 118.55–253.70	25.75 ± 2.78	98.47	0.70	37.01	100.00
	Xinjiang	384	224.28, 165.62–282.11	28.17 ± 5.30	94.53	0.46	7.83	100.00
	Total	1493	187.30, 122.80–258.08	26.07 ± 4.28	93.85	0.85	29.71	100.00

^a^MUIC, median urinary iodine concentration.

^b^Consumption rate, the number of subjects receiving qualified iodized salt (iodine preparations or iodine-rich food) / the total number of subjects surveyed.

^c^Iodine supplement rate, the proportion of subjects who take iodized salt, iodine supplement preparations or iodine-rich foods.

^d^Qualified iodized salt, salt samples with iodine of 18-33mg/kg.

### Iodine supplement measures

The iodine supplement measures of pregnant women and adults were significantly different (*p* < 0.01, **Table 3**). As for pregnant women, IS (61.6%) and ISF (33.4%) were more common iodine supplement measures, followed by ISP and ISFP (both of them were 1.7%), and the lowest proportion of iodine supplement was IF (1.6%). In Fujian, IS (61.8%) was still the main way to supplement iodine; the next was ISF (23.2%). Similar to pregnant women, IS was the main way to supplement iodine for adults, accounting for 67.7%, followed by ISF (30.3%) and IF (1.2%). From the point of each province, the largest proportion of iodine supplement in Fujian was ISF (56.5%), and the proportion of iodine supplement with IF was 3.6%, which was higher than that of other provinces (all of them were 0.5%). Xinjiang accounted for the largest proportion of iodine supplement with IS, which was 91.8%.

**Table 3:** The iodine supplement measure of adults and pregnant women.

Type	District	Number	Iodine Supplement Measures^*^ (N/%)				
			IS^a^	ISF^b^	IF^c^	ISP^d^	ISFP^e^
Pregnant women	Fujian	319	74/23.2	197/61.8	20/6.3	8/2.4	20/6.3
	Shanxi	852	576/67.6	263/30.9	7/0.8	3/0.35	3/0.35
	Yunnan	409	275/67.2	130/31.8	1/0.2	3/0.8	0/0.0
	Xinjiang	403	297/73.7	72/17.9	4/1.0	19/4.7	11/2.7
	Total	1983	1,222/61.6	662/33.4	32/1.6	33/1.7	34/1.7
Adults	Fujian	329	130/39.5	186/56.5	12/3.6	0/0.0	1/0.3
	Shanxi	395	297/75.2	89/22.5	2/0.5	5/1.3	2/0.5
	Yunnan	428	264/61.7	158/37.0	2/0.5	3/0.7	1/0.1
	Xinjiang	366	336/91.8	27/7.4	2/0.5	1/0.3	0/0.0
	Total	1518	1,027/67.7	460/30.3	18/1.2	9/0.6	4/0.3

^*^ represented the iodine supplement measures of pregnant women and adults were different and P < 0.01.

^a^IS, Iodine Salt.

^b^ISF, Iodine Salt + Iodine rich Food.

^c^IF, Iodine rich Food, refers to eating more than once per month of 50 grams or above, wet weight of kelp, seaweed etc. and not eating iodized salt.

^d^ISP, Iodine Salt + Iodine Preparation.

^e^ISFP, Iodine Salt + Iodine rich Food + Iodine Preparation.

### The effect of iodine supplement measures on iodine nutrition

Through the analysis of non-parametric rank test (**Table 4**), pregnant women’s MUIC of supplement iodine with ISFP group (229.92 μg/L) was significantly higher than that of IS (168.90 μg/L) and ISP (175.56 μg/L), and pregnant women supplement iodine with IF (115.91 μg/L) and ISF (149.69 μg/L) had the lowest level, which illustrated that ISFP was an effective measure to improve iodine nutrition; whereas for adults, all of the MUICs of ISFP (202.40 μg/L), IS (192.18 μg/L), and ISP (209.95 μg/L) were higher than that of IF (150.50 μg/L) and ISF (171.30 μg/L). In addition, contrast to pregnant women supplement iodine with IS, supplement iodine with ISFP could decreased the threat of deficiency iodine nutrition (*p* < 0.05), whereas for adults, different iodine supplement measures had similar effects on iodine nutrition.

**Table 4:** The effect of iodine supplement measures on iodine nutrition.

Type	Classification	MUIC(μg/L)Median (IQR)	Normal Group and Low Group				Normal Group and High Group			
			OR	CI		*P*	OR	CI		*P*
Pregnant women	IS	168.90	Ref	Ref	Ref	Ref	Ref	Ref	Ref	Ref
	ISF	149.69	1.229	0.986	1.530	0.066	0.807	0.617	1.056	0.118
	IF	115.91	1.735	0.752	4.005	0.197	0.629	0.188	2.109	0.453
	ISP	175.56	0.950	0.412	2.189	0.904	1.258	0.517	3.062	0.613
	ISFP	229.92	0.292	0.112	0.760	0.012	1.090	0.511	2.326	0.823
	*p*	0.000 *Post hoc post hoc*test: ISFP > IS, ISP > ISF, IF								
Adults	IS	192.18	Ref	Ref	Ref	Ref	Ref	Ref	Ref	Ref
	ISF	171.30	1.318	0.988	1.759	0.061	0.805	0.576	1.124	0.202
	IF	150.50	0.671	0.149	3.026	0.603	0.344	0.044	2.662	0.307
	ISP	209.95	4.024	0.805	20.120	0.090	2.749	0.456	16.591	0.270
	ISFP	202.40	2.012	0.181	22.325	0.569	2.062	0.186	22.882	0.556
	*p*	0.008 *Post hoc post hoc*test: ISFP, IS, ISP > ISF, IF								

### Contribution of dietary iodine

Whether pregnant women (39.3%) or adults (56.9%), IS was still the main measure to supplement iodine (**Table 5**). From the perspective of pregnant women, the median iodine intake from drinking water in the four provinces was 3.5 μg/day, with a contribution rate of 1.4%; the average intake rates of iodine from food, IS, and preparations were 86.5, 107.2, and 75.3 μg/day, respectively (the data were mainly affected by the iodine supplement of Xinjiang, and the average iodine of preparation in Xinjiang was 281.6 μg/day with the contribution rate of 57.7%). To exclude the overall impact of lipiodol taken by pregnant women in Xinjiang, the data from other three provinces were analyzed, and the contribution rate of preparations was 3.1%; iodine intake from food and IS were 80.2 μg/day (40.5%) and 108.0 μg/day (54.5%), respectively. As for adults, different from Fujian, Shanxi, and Yunnan, food was the main source of dietary iodine intake in Xinjiang, and the daily iodine intake from food was 155.8 μg, accounting for 56.8%, followed by IS (116.9 μg/day, accounting for 42.6%).

**Table 5:** The iodine supplement contribution^a^ by different sources of adults and pregnant women.

Type	District	Number	FoodMean (μg/day)	Contribution Rate (%)	IodizedSaltMean (μg/day)	Contribution Rate (%)	DrinkingWaterMedian (μg/day)	Contribution Rate (%)	PreparationsMean (μg/day)	Contribution Rate (%)
Pregnant women	Fujian	306	98.6	41.1	121.3	50.6	2.4	1.0	17.5	7.3
	Shanxi	865	82.8	43.3	102.1	53.3	5.1	2.7	1.4	0.7
	Yunnan	408	65.2	38.8	100.1	59.5	2.9	1.7	−	−
	Xinjiang	367	98.4	20.2	104.4	20.6	3.1	1.5	281.6	57.7
	Total (not including Xinjiang)	1,579	80.2	40.5	108.0	54.5	3.8	1.9	6.0	3.1
	Total (including Xinjiang)	1,946	86.5	31.7	107.2	39.3	3.5	1.4	75.3	27.6
Adults	Fujian	407	117.6	41.2	162.7	57.0	5.0	1.8	−	−
	Shanxi	400	67.0	27.0	167.9	67.8	12.8	5.2	−	−
	Yunnan	435	60.4	32.7	119.2	64.5	5.2	2.8	−	−
	Xinjiang	436	155.8	56.8	116.9	42.6	1.7	0.6	−	−
	Total	1,678	100.6	40.7	140.8	56.9	6.0	2.4	−	−

^a^Iodine supplement contribution, iodine intake in drinking water, food, iodine preparations or iodized salt / all amounts of iodine intake.

### The consumption frequency of food (iodine contain ≥ 100 μg/100 g)

There were many foods with similar consumption frequency for pregnant women and adults (**Table 6**), in which “never” accounted the largest percentage. For marine fish, only 2.3% of pregnant women ate at least once a day, whereas adults accounted for 6.1%; as for kelp or undaria and nori, pregnant women accounted for 1.4% and 1.6% of consumption at least once a day, respectively, which was higher than 0.3% and 0.4% for adults; in addition, pregnant women ate more sea sedge and seaweed snacks than adults; finally, for picked vegetables, the consumption frequency of adults was higher than that of pregnant women, and the percentages of adults and pregnant women who chose “never” were 43.4% and 64.1%, respectively.

**Table 6:** The consumption frequency of food (iodine contain ≥ 100 μg/100 g).

Type	Variety	Classification	Consumption Frequency (N/%)				
			≥ 1 time/day	1–6 times/week	1–3 times/month	1–5 times/6 months	Never
Pregnant women	Aquatic products	Marine fish (Tanichthys albonubes, Turbot, cutlassfish, etc.)	41/2.3	252/14.0	330/18.3	97/5.4	1,085/60.0
		Shrimp, dried shrimp, crab, etc.	10/0.6	164/9.1	499/27.6	132/7.3	1,001/55.4
		Kelp, undaria (fresh)	26/1.4	160/8.9	610/33.8	145/8.0	865/47.9
		Nori (dried)	29/1.6	159/8.8	436/24.1	122/6.8	1,060/58.7
		Sea sedge (po-li, joyful-time, etc.)	11/0.6	50/2.8	95/5.3	105/5.8	1,544/85.5
	Snack	Seaweed or seaweed snack	26/1.4	72/4.0	113/6.3	104/5.8	1,490/82.5
	Condiments	Ginger powder	260/14.4	151/8.4	67/3.7	27/1.5	1,300/72.0
		Shrimp paste	2/0.1	9/0.5	17/0.9	23/1.3	1,754/97.2
		Picked vegetables	69/3.8	271/15.0	206/11.4	103/5.7	1,156/64.1
		Chicken essence	390/21.6	117/6.5	72/4.0	41/2.3	1,185/65.6
Adults	Aquatic products	Marine fish (Tanichthys albonubes, Turbot, cutlassfish, etc.)	98/6.1	205/12.8	247/15.5	115/7.2	933/58.4
		Shrimp, dried shrimp, crab, etc.	14/0.9	195/12.2	355/22.2	177/11.1	857/53.6
		Kelp, undaria (fresh)	5/0.3	136/8.5	567/35.5	248/15.5	642/40.2
		Nori (dried)	6/0.4	144/9.0	439/27.5	140/8.8	869/54.3
		Sea sedge (po-li, joyful-time, etc.)	2/0.1	9/0.6	50/3.1	111/6.9	1,426/89.3
	Snack	Seaweed or seaweed snack	7/0.4	11/0.7	51/3.2	123/7.7	1,405/88.0
	Condiments	Ginger powder	195/12.2	238/14.9	64/4.0	26/1.6	1,075/67.3
		Shrimp paste	1/0.1	8/0.5	23/1.4	30/1.9	1,536/96.1
		Picked vegetables	87/5.4	326/20.4	325/20.3	167/10.5	693/43.4
		Chicken essence	394/24.7	153/9.6	56/3.5	53/3.3	942/58.9

### Investigation of dietary salt intake

**Figure 2:**
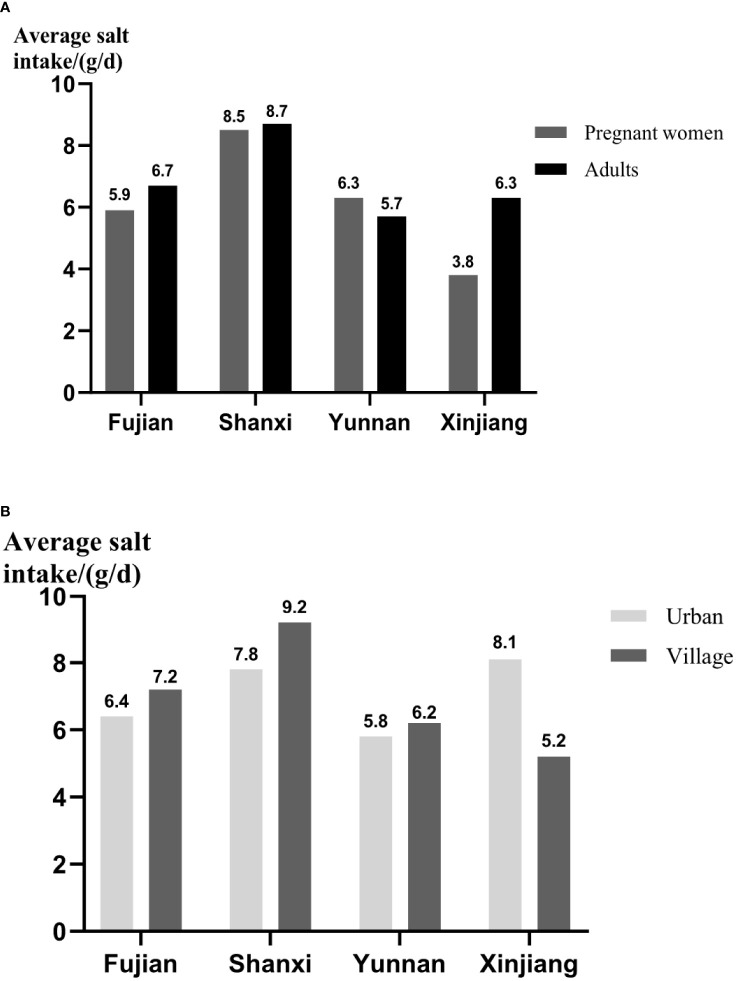
The dietary salt intake of different populations and areas. **(A)** The comparison of salt intake between pregnant women and adults. **(B)** The comparison of salt intake between adults in urban and village. The daily average intake of salt was presented as means ± SD.

By comparing the IS intake of pregnant women in different provinces (**Figure 2A**), it was found that Shanxi had more salt intake, which was 8.5 g/day, and the next was Yunnan with 6.3 g/day. As for adults, Shanxi (8.7 g/day) and Yunnan (5.7 g/day), respectively, had the highest and lowest dietary salt intake. In addition to pregnant women and adults in Yunnan, the dietary salt intake of the former was less than that of the latter, especially those in Xinjiang. Moreover, through the comparison of habitation (urban/village, **Figure 2B**), except Xinjiang, the adults in village had the more amount of salt intake than urban, and the salt intake of rural residents in Shanxi was as high as 9.2 g/day. Whereas in Xinjiang, the salt intake of urban residents was 8.1 g/day, whereas that of rural residents was 5.2 g/day.

### The history of thyroid diseases

**Figure 3:**
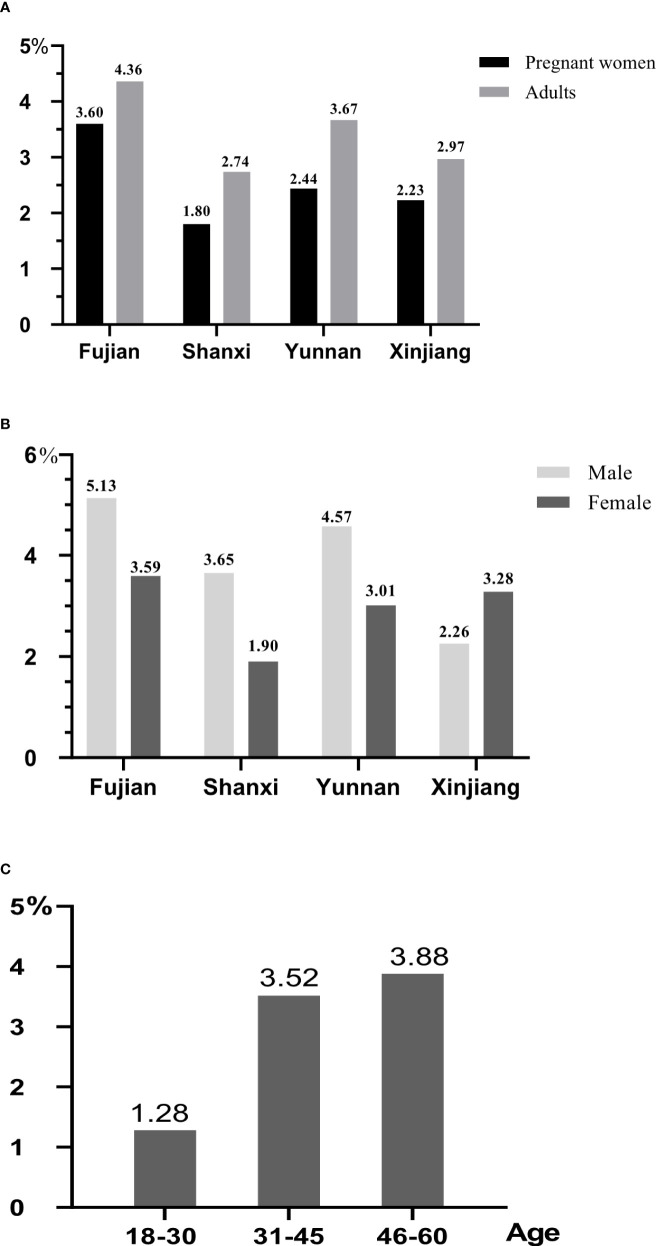
The history of thyroid diseases for different populations. All data were presented as percentage. **(A)** The comparison of history of thyroid diseases between adults and pregnant women. **(B)** The comparison of history of thyroid diseases between male and female. **(C)** The comparison of history of thyroid diseases for adults in different age.

Comparing the history of thyroid diseases between pregnant women before conception and adults (**Figure 3A**), it was found that adults’ history of thyroid diseases were higher than that of pregnant women in four provinces. The history of thyroid diseases for pregnant women (3.60%) and adults (4.36%) in Fujian had the highest rates, the next was those in Yunnan. The prevalence rates of thyroid diseases for men in Fujian, Shanxi, and Yunnan were 5.13%, 3.65%, and 4.57% (**Figure 3B**), respectively, which were higher than that for women. However, Xinjiang was just reversed, and the prevalence of pregnant women (3.28%) was higher than men (2.26%). Through analyzing the history of thyroid diseases in different age, it was found that it increased with the change of age (**Figure 3C**), and the subjects with the age between 46 and 60 years old had the highest rate (3.88%), whereas those with the age between 18 and 30 years old only had the rate of 1.28%.

### The prevalence of thyroid diseases for pregnant women

**Figure 4:**
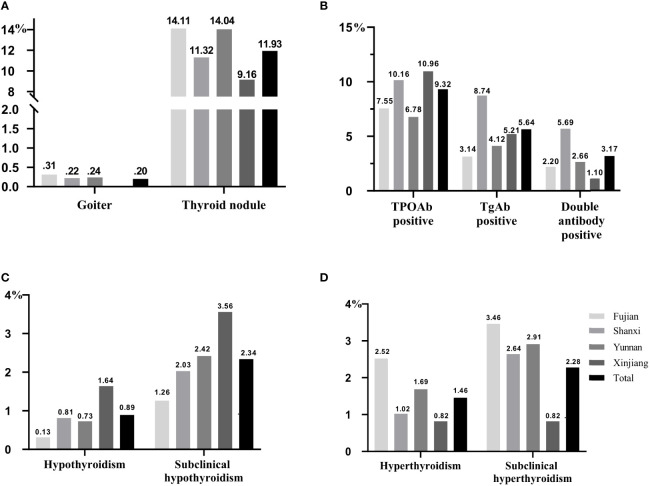
The prevalence of thyroid diseases in pregnant women. All data were presented as prevalence rate. **(A)** The prevalence of thyroid goiter and nodule. **(B)** The prevalence of antibody positive. **(C)** The prevalence of hypothyroidism and subclinical hypothyroidism. **(D)** The prevalence of hyperthyroidism and subclinical hyperthyroidism.

Among the thyroid diseases of pregnant women, goiter had the lowest prevalence (**Figure 4A**), and the highest prevalent disease was thyroid nodules, accounting for 11.93%, followed by TPOAb positive (9.32%, **Figure 4B**). Subclinical hypothyroidism (2.34%) had the highest prevalence among the thyroid function related diseases (**Figure 4C**), followed by subclinical hyperthyroidism (2.28%, **Figure 4D**). In provincial level, first, the prevalence rates of thyroid nodules in Fujian and Yunnan were high with 14.11% and 14.04%, respectively. Second, Shanxi had the highest prevalence of TgAb positive (8.74%) and double antibody positive (5.69%); however, the highest prevalence of TPOAb positive was 10.96% in Xinjiang, followed by 10.16% in Shanxi. Finally, the prevalence of hypothyroidism (1.64%) and subclinical hypothyroidism (3.56%) in Xinjiang were the highest, and as for hyperthyroidism and subclinical hyperthyroidism, Fujian had the highest prevalence, 2.52% and 3.46%, separately, followed by Yunnan (1.69% and 2.91%), and the their prevalence in Xinjiang was the lowest, both of them was 0.82%.

### Factors affecting thyroid diseases of pregnant women

**Figure 5:**
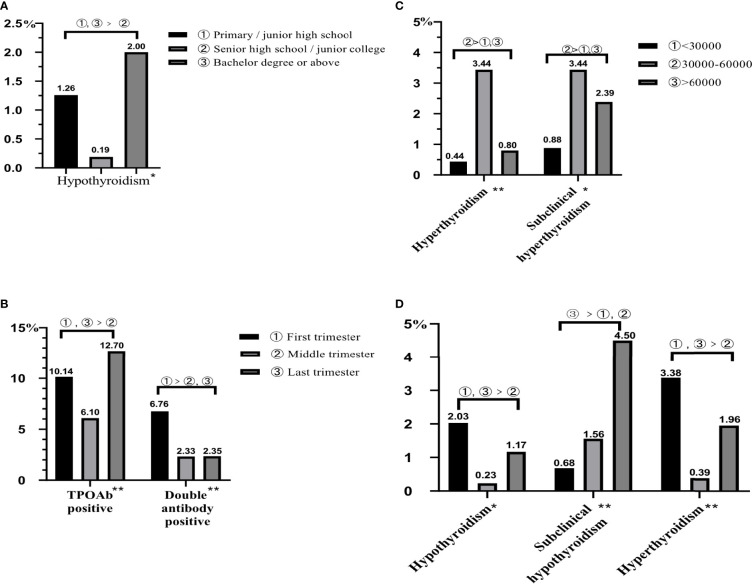
The influencing factors of thyroid diseases in pregnant women. All data were presented as rate. **(A)** The influence of degree of education on hypothyroidism. **(B)** The influence of gestation on TPOAb positive and double antibody positive. **(C)** The influence of family annual income on hyperthyroidism and subclinical hyperthyroidism. **(D)** The influence of gestation on hypothyroidism, subclinical hypothyroidism, and hyperthyroidism. *represents *P <* 0.05; **represents *P <* 0.01.

Analyzing factors that might influence the pregnant women’s prevalence of thyroid diseases, pregnant women living in urban had lower prevalence of TgAb positive (4.83%, **Table 7**). As for gestation, it would have influences on many thyroid diseases, such as hypothyroidism, subclinical hypothyroidism, hyperthyroidism, TPOAb positive, and double antibody positive. The prevalence of hypothyroidism, hyperthyroidism, and TPOAb positive in the first and last trimesters was higher than those in the middle trimester (0.23%, 0.39%, and 6.10%; **Figures 5B**, **D**), and the prevalence of subclinical hypothyroidism in the last trimester was the highest (4.50%); moreover, the prevalence of double antibody positive in the first trimester was the highest (6.76%). In addition, degree of education also had an effect on thyroid diseases; pregnant women of senior high school or junior college had the lowest rate of hypothyroidism (0.19%, **Figure 5A**). In terms of household annual income (**Figure 5C**), pregnant women with less or more family annual income had lower prevalence of hyperthyroidism and subclinical hyperthyroidism.

**Table 7:** The influencing factor of thyroid diseases for pregnant women.

Indication	Classification	Prevalence rate (%)								
		Hypothyroidism^a^	Sub clinical hypothyroidism^b^	Hyperthyroidism^c^	Sub clinical Hyperthyroidism^d^	TPOAb Positive^e^	TgAb Positive^f^	Double Antibody Positive^g^	Goiter^h^	Thyroid Nodule^i^
Gestation	First trimester	2.03	0.68	3.38	1.69	10.14	8.11	6.76	0.00	10.93
	Middle trimester	0.23	1.56	0.39	2.20	6.10	5.32	2.33	0.11	13.99
	Last trimester	1.17	4.50	1.96	2.74	12.70	4.36	2.35	0.45	11.45
	*P*	0.011	0.000	0.001	0.629	0.000	0.111	0.001	0.324	0.184
Habitation	Village	0.75	2.75	1.00	1.87	9.49	6.87	3.50	0.39	16.01
	Urban	1.42	1.42	2.27	2.56	10.51	4.83	3.13	0.00	14.33
	*P*	0.221	0.121	0.080	0.293	0.330	0.025	0.451	0.334	0.264
Degree of education	Primary/junior high school	1.26	2.73	1.05	1.89	10.71	6.30	2.94	0.00	13.95
	Senior high school/junior college	0.19	1.34	1.92	2.87	9.20	5.94	3.45	0.59	17.47
	Bachelor degree or above	2.00	4.00	0.67	0.00	12.00	6.00	2.67	0.00	13.91
	*P*	0.027	0.225	0.393	0.075	0.564	0.985	0.867	0.363	0.258
Annual iocome	<30,000	0.22	2.41	0.44	0.88	10.72	7.00	3.94	0.22	15.35
	30,000–60,000	1.88	2.19	3.44	3.44	12.50	6.88	4.06	0.00	14.79
	>60,000	0.80	2.39	0.80	2.39	7.45	4.52	1.86	0.55	16.09
	*P*	0.053	0.988	0.001	0.041	0.081	0.271	0.171	0.391	0.888
Drinking water type	Tap water	0.57	2.87	1.95	2.07	10.46	5.98	3.45	0.25	10.60
	Rests	1.18	1.77	0.88	2.65	8.26	5.60	2.65	0.00	13.68
	*P*	0.115	0.106	0.063	0.227	0.084	0.422	0.229	0.220	0.023

^a^Hypothyroidism, TSH is greater than the upper limit of the normal range, and FT4 is less than the lower limit of the normal range.

^b^Subclinical hypothyroidism, TSH is greater than the upper limit of the normal range, and FT4 is within the normal range.

^c^Hyperthyroidism, TSH is less than the lower limit of the normal range, and FT4 is greater than the upper limit of the normal range.

^d^Subclinical hyperthyroidism, TSH is less than the lower limit of the normal range, and FT4 is within the normal range

^e^TPOAb positive, TPOAb is greater than the upper limit of the normal range.

^f^TgAb positive, TgAb is greater than the upper limit of the normal range.

^g^Double Antibody positive, both TPOAb and TgAb are greater than the upper limit of the normal range.

^h^Goiter, thyroid volume is beyond the upper limit of the normal range.

^i^Thyroid nodule, pathological changes caused by local abnormal growth of thyroid cells.

### Investigation of dining place of adults

**Figure 6:**
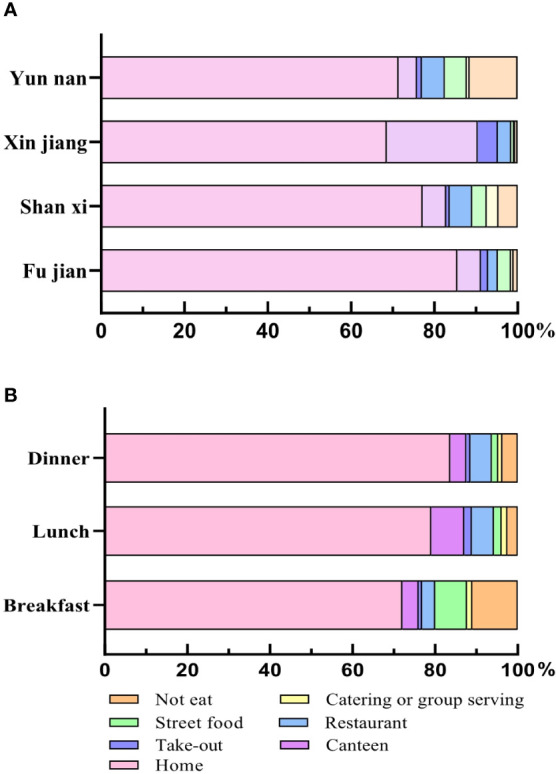
The dining place for different provinces and meals in adults. **(A)** The dining place of adults in four provinces. **(B)** The dining place of three meals. All data were presented as percentage.

Adults had the largest proportion of meals at home (**Figure 6A**); Fujian had the highest percentage of 85.54%. In Fujian, Shanxi, and Xinjiang, the next one was in canteens, accounting for 5.68%, 5.64% and 21.79%, respectively. In addition, through the analysis of three meals a day (**Figure 6B**), adults had the larger proportion of breakfast at home (72.10%) and street food (7.73%). Different from breakfast, the most common dining locations for lunch and dinner were at home, canteen, and restaurant.

## Discussion

Through this study, first, it was found that the iodine nutrition of pregnant women was lower than that of adults for the same area; in addition, from the view of iodine supplement measures, IS was still the main measure for people, the difference between pregnant women and adults was mainly about iodine preparations, and the consumption rate of it in pregnant women was higher than adults; finally, for the effects of different iodine supplement measures on iodine nutrition, it was found that iodine supplement with ISFP could effectively improve the iodine nutrition of pregnant women, followed by IS and ISP, whereas for adults, the effects of ISFP, IS, and ISP on iodine nutrition were similar.

Adequate dietary iodine intake was essential for populations, especially pregnant women (35). According to the investigation and analysis, the urinary iodine level of pregnant women in China was close to the lower limit of adequate iodine status (150 μg/L); and the MUIC of adults was 187.30 μg/L, which was within the recommended normal range of 100–200 μg/L (36). The iodine nutrition of pregnant women and adults were adequate, and gender was irrelevant to adults’ iodine nutrition, which was consistent with the research results of Yang LC et al. (37, 38); however, at the provincial level, the MUICs of pregnant women in Fujian and Yunnan were both below 150μg/L, defined as ID (39), where more iodine intake was required, for example, increasing the supplement of IS, iodine-rich food, and iodine preparations.

Choosing appropriate iodine supplement measure promotes the healthy development of pregnant women and adults. Consistent with the study of König et al. (40), IS was still the main measure to supplement iodine for people. The rate of pregnant women supplemented iodine with preparations was higher than adults, which might be due to the increased iodine requirements during pregnancy; ID *in utero* damaged the fetus’ developing brain, leading to the loss of millions of IQ points globally (41), and three trials involving 543 pregnant women found that those who received iodine supplement were less likely to develop the unwanted effect of hyperthyroidism after giving birth (42). In addition, although pregnant women and adults’ iodine nutrition reached the recommendation of WHO (43), the iodine intake of pregnant women through dietary was lower than that of adults, and different from adults, the iodine intake of pregnant women was lower than the recommended iodine intake (RNI, 230 μg/day) (44) in the case of excluding Xinjiang, which might be partly due to the vomiting reaction that affected the appetite of pregnant women and so on. In the study, it was found that ISFP was a more effective measure for pregnant women and adults to improve iodine nutrition, especially for pregnant women; however, Schiller et al. (45) found that UIC was higher in women consuming iodine preparations, which was consistent with this study, but they also found that no association between UIC and dietary iodine content or water iodine, and it might because that MUIC was used to measure iodine nutrition in this study. Pregnant women should pay attention to not only the iodine supplement but also the source and amount of it. At the same time, excess iodine intake should also be avoided.

IS was the main source of dietary iodine in many countries (40), but the intake of it was closely related to sodium intake. For reducing cardiovascular mortality worldwide, the WHO has recommended reducing salt intake to less than 5 g per day (<2,000 mg sodium/day) in adults (46), which might have an effect on iodine nutrition of population. However, except for pregnant women in Xinjiang, the salt intake was higher than the recommendation of WHO in this study. Elizabeth et al. thought that coordinating interventions designed to reduce population sodium intake with salt iodization programs was essential to maintain adequate levels of iodine nutrition as salt intake declines (47).

This study found that the most common thyroid diseases in pregnant women were thyroid nodule, subclinical hypothyroidism, and positive antibody, especially TPOAb positive, which was consistent with other investigation (48, 49); however, the prevalence of subclinical hyperthyroidism was higher than other study (50), which might be the result of different reference of the thyroid function. Alexander et al. found that the immune response caused by the binding of thyroid peroxidase (TPO) to TPOAb could damage thyroid cells and further lead to hypothyroidism (51). Combined with urinary iodine of pregnant women, it could be seen that both deficiency and excessiveness in the iodine nutrition would affect the thyroid function of pregnant women (52).

Many factors would affect the prevalence of thyroid diseases in pregnant women, especially gestation. Glinoer (53) found that the hormonal changes and metabolic demands during pregnancy result in alterations in the biochemical parameters of thyroid function. In addition, habitation, household annual income, and degree of education also had an effect on the thyroid diseases, which was possibly because of their influence on iodine nutrition, and degree of education may affect the awareness of IDD and the prevention of IS. Taylor et al. illustrated that iodine nutrition was a key determinant of thyroid diseases, and the habitation, household income, and so on would affect the quality and diversity of pregnant women’s diet (54), but the exact reasons for this need further study.

In this study, sufficient samples were investigated, multi-stage sampling was adopted, and the sample was very representative. The food frequency table covered main kinds of food containing iodine; the information collected by the questionnaire was sufficient. However, food weighing method was not used in this food survey, and the diet survey might have bias, although every investigator had been trained to ensure the accuracy of the survey results. Moreover, the dining place of pregnant women and the thyroid function of adults were not investigated.

## Conclusion

The iodine nutrition of pregnant women and adults was adequate in the four provinces; the largest difference of iodine supplement between them was the using of iodine preparations and the amount of dietary iodine intake; and the dietary iodine intake of pregnant women was less than the RNI, which illustrated that it was essential for them to increase the intake of iodine. ISFP was an effective measure for population to supplement iodine, especially for pregnant women. Many factors would affect thyroid disease prevalence in pregnant women, such as habitation (urban/rural) and gestation; those in the first and last trimesters should pay more attention to the measurement and amount of iodine supplement. Finally, although we need to decrease the consumption of salt, it is important to coordinate the relationship between iodine nutrition and low sodium diet.

## Data availability statement

The datasets presented in this article are not readily available because it involves personal information, it is not convenient to disclose. Requests to access the datasets should be directed to the corresponding author.

## Ethics statement

The studies involving human participants were reviewed and approved by the Ethics Committee of Harbin Medical University (20190502). The patients/participants provided their written informed consent to participate in this study.

## Author contributions

DS and PL designed research; YD, LL, TQ, WC, and MZ carried out research; RS and LF analyzed data; and RS wrote the paper. RS and LF had primary responsibility for final content. All authors read and approved the final manuscript.

## Funding

This research was supported by National Natural Science Foundation, 81773370; the 2019 China Hygiene and Health Standard Project, 20190502; HMU Marshal Initiative Funding, HMUMIF-21015; and National Nature Science Foundation, 81830098.

## Acknowledgments

We thank all participants in this study and the staff in the Provincial Center for Disease Control and Prevention or Institute of Endemic Disease Prevention and Control of Xinjiang, Fujian, Yunnan, and Shanxi.

## Conflict of interest

The authors declare that the research was conducted in the absence of any commercial or financial relationships that could be construed as a potential conflict of interest.

## Publisher’s note

All claims expressed in this article are solely those of the authors and do not necessarily represent those of their affiliated organizations, or those of the publisher, the editors and the reviewers. Any product that may be evaluated in this article, or claim that may be made by its manufacturer, is not guaranteed or endorsed by the publisher.
